# Pivotal neuroinflammatory and therapeutic role of high mobility group box 1 in ischemic stroke

**DOI:** 10.1042/BSR20171104

**Published:** 2017-12-05

**Authors:** Seidu A. Richard, Marian Sackey, Zhaoliang Su, Huaxi Xu

**Affiliations:** 1 Department of Immunology, Jiangsu University, 301 Xuefu Road, Zhenjiang 212013, P.R. China; 2 Department of Surgery, Volta Regional Hospital, P.O. Box MA-374, Ho, Ghana-West Africa; 3 Department of Pharmacy, Volta Regional Hospital, P.O. Box MA-374, Ho, Ghana-West Africa

**Keywords:** atherosclerosis, angiogenesis, HMGB1, Hypoglycaemia, Ischemic stroke, oedema

## Abstract

Stroke is a major cause of mortality and disability worldwide. Stroke is a frequent and severe neurovascular disorder. The main cause of stroke is atherosclerosis, and the most common risk factor for atherosclerosis is hypertension. Therefore, prevention and treatment of stroke are crucial issues in humans. High mobility group box 1 (HMGB1) is non-histone nuclear protein that is currently one of the crucial proinflammatory alarmins in ischemic stroke (IS). It is instantly released from necrotic cells in the ischemic core and activates an early inflammatory response. HMGB1 may signal via its putative receptors, such as receptor for advanced glycation end products (RAGE), toll-like receptors (TLRs) as well as matrix metalloproteinase (MMP) enzymes during IS. These receptors are expressed in brain cells. Additionally, brain-released HMGB1 can be redox modified in the circulation and activate peripheral immune cells. The role of HMGB1 may be more complex. HMGB1 possesses beneficial actions, such as endothelial activation, enhancement of neurite outgrowth, and neuronal survival. HMGB1 may also provide a novel link for brain-immune communication leading to post-stroke immunomodulation. Therefore, HMGB1 is new promising therapeutic intervention aimed at promoting neurovascular repair and remodeling after stroke. In this review, we look at the mechanisms of secretion of HMGB1, the role of receptors, MMP enzymes, hypoglycemia, atherosclerosis, edema, angiogenesis as well as neuroimmunological reactions and post-ischemic brain recovery in IS. We also outline therapeutic roles of HMGB1 in IS.

## Introduction

Ischemic stroke (IS) occurs when a cerebral blood vessel is occluded. This disrupts the cerebral supply with nutrients and oxygen leading to permanent necrotic brain tissue destruction in the affected area of the respective terminal artery. This focal brain damage can result in various acute neurological deficits [1,2]. It is estimated that the overall costs of stroke care will account for 6.2% of the total burden of illness by 2020 [2,3]. Hypertension (HT), hyperlipidemia (HL), and diabetes mellitus (DM) are the most common risk factors for atherosclerosis, which is the key trigger of stroke [2,4].

High mobility group box 1 (HMGB1) is non-histone nuclear protein that is currently one of the crucial proinflammatory alarmins in IS [1]. It has been indicated that the secretion of endogenous alarmins also known as danger-associated molecular patterns (DAMPs) from necrotic cerebral cells may be one of the core initiators of the inflammatory cascade. It is now clear that DAMPs releases from the necerotic tissues initiate local inflammary response in the infart core in ischemic brain injury but when released into the blood,it may signal peripheral immune cells.[1,5]. Several studies have confirmed HMGB1 as one of the DAMPs that has been shown to be involved in ischemic brain injury [6–9]. It is usually confined in cell nuclei in the normal brain, translocates into the cytosol, and is secreted into the extracellular compartment under ischemic conditions. It has been reported that intracellular HMGB1 plays a critical role in activation and regulation of basal transcriptional machinery [8,10,11] and energy homeostasis. Studies have shown that the extracellular secretion of HMGB1 is observed within 2–4 h after ischemia–reperfusion, increases vascular permeability, and promotes blood–brain barrier (BBB) breakdown [9,12]. Zhang et al. [12] indicated that the neutralization of extracellular HMGB1 prevented the BBB permeability and curtails the infarct volume in mice and rats. Therefore, HMGB1 is a fundamental DAMP in the hyperacute phase of ischemic brain injury. Qiu et al. [13] also indicated that the extracellular release of HMGB1 dwindles within 12 h after ischemia–reperfusion.

In this review, we look at the role of the redox form of HMGB1, the mechanisms of release of HMGB1 in the early phase of IS as well as the receptors via which HMGB1 interacts. We also looked at the role of HMGB1 in atherosclerosis, angiogenesis, edema, hyperglycemia as well as its neuroimmunological properties and post-ischemic brain recovery. We suggest that more research with both animal and human models is needed to further confirm that HMGB1 has therapeutic potential since initial studies proved that it promotes neurovascular repair and remodeling after stroke.

## The redox state of HMGB1 in strokes

HMGB1 is a non-histone DNA-binding protein of 215 amino acid residues arranged in three domains that include A box and B box domains arranged in an L-shape and a 30-amino acid long C-terminal tail [14–17]. Muhammad et al. [18] indicated that HMGB1 reduces ischemic brain injury in a mouse model of experimental stroke through Box A which they discovered as antagonist of inflammation during IS.Li et al revealed that the cytokine-inducing activity of disulphide HMGB1 is located on B the B-box and it is made up of Cys^106^ residues. However, antibody-mediated targetting role of B box is still not known, so we suggest further focus on this role.

It has been shown that the redox modification of HMGB1 occurs at three cysteine residues at positions 23, 45, and 106, which regulate the functional properties of HMGB1. Increased levels of reactive oxygen species (ROS) lead to the formation of disulphide bonds between cysteine 23 and 45 [1]. These bonds have proven to induce nuclear factor κ-light-chain-enhancer of activated B cells (NF-κB) translocation to the nucleus and tumor necrosis factor alpha (TNF-α) synthesis by macrophages [1,20]. Studies have further shown that all cysteine residues in HMGB1 can also be oxidized by the sustained ROS and nitrogen species (RNS) secretion from the mitochondria of apoptotic cells [1]. This kind of terminally oxidized HMGB1 is known as sulphonyl HMGB1, which gives anti-inflammatory signals and curtails excessive inflammatory activity [1,21]. HMGB1 can be modified into a reduced or oxidized form exerting specific cellular functions [1].

## HMGB1 release in the acute phase after stroke in animal models

Cerebral blood vessel occlusion in stroke causes a rapid necrosis of the affected brain region. Brain resident microglia and astrocytes in reaction to ischemic injury, get stimulated and release a storm of ROS and RNS and proinflammatory cytokines that cause secondary damage to the infarct area [1,22]. The onsite recruitment of peripheral immune cells that further exaggerate the oxidative stress through release of ROS and RNS is accompanied by extensive inflammation of the ischemic brain tissue. The inflammatory processes build a highly oxidative circumstance in the ischemic brain. Essentially, the action and function of several proteins is controlled by oxidation of cysteine, tyrosine, and methionine residues [1].

HMGB1 is expressed as a soluble peptide with proinflammatory cytokine function in reaction to a cell activation stimulus or passively after necrotic cell death. It synergizes with other proinflammatory molecules and cascades in the beginning of neuroinflammation [1]. Necrotic cells secrete hypoacetylated HMGB1 and signal innate immune cells to respond to the tissue injury that leads to the recruitment of mononuclear cells to the tissue injury and stimulate an inflammatory milieu [1,23]. HMGB1 immediately (i.e. within 1 h after ischemia onset) translocates from nuclei to the cytoplasm in neurones in the ischemic brain. Its release in activated microglia, astrocytes, and blood vessels is amplified 2 days after middle cerebral artery occlusion (MCAO) in rats [13,24]. *In vivo* knockdown of HMGB1 release by intrastriatal injection of HMGB1-shRNA 24 h before MCAO decreases infarct size and microglia activation, signifying a neurotoxic and proinflammatory role of HMGB1 after stroke [1,6].

HMGB1 expressed from necrotic tissue can induce inflammatory cytokine release in the infarcted brain. It has been demonstrated that stereotactical injection of fully reduced recombinant HMGB1 into the parietal cortex of mice forces the mRNA to release inducible nitric oxide synthase (iNOS) and interleukin (IL)-1β (IL-1β) after 24 h [1,25]. Also *in vitro* cultures of mouse brain derived glial cells indicated amplified release of iNOS, IL-1β, Cyclooxygenase-2 (COX-2), and TNF-α after treatment with recombinant HMGB1 [25], signifying an activation of glial cells by HMGB1 that was potentially secreted by necrotic neurones. It is also clear that the ‘maturation’ of the HMGB1 in brain parenchyma and blood toward a hyperacetylated state can be detected within 24 h after brain insult [26].

## HMGB1 release in the acute phase after stroke in human models or patients

Studies have indicated that HMGB1 levels in the blood of stroke patients are higher as compared with matched healthy controls [27,28]. HMGB1 concentrations remain significantly elevated for a prolonged time period in symptomatic patients. Schulze et al. [29] demonstrated that in acute IS patients presented with elevated plasma concentrations of HMGB1 that persistently rise for 30 days after the primary insult and associated with a number of circulating leukocytes. They further indicated that increased HMGB1 levels have incoherent correlation with infarct size and location, since higher HMGB1 levels are observed in patients with more severe cerebral lesions [29].

Furthermore, the levels of HMGB1 in patients with stroke remain significantly elevated than in control subjects up to 14 days after the ischemic event while levels of the natural inhibitors of HMGB1, soluble receptor for advanced glycation end products (sRAGE) and endogenous secretory RAGE (esRAGE), remain indistinguishable from control subjects within 48 h following stroke. In patients with stroke, HMGB1 levels are adequately associated with IL-6 levels but not with the magnitude of brain tissue damage as evaluated by CT morphometry. Also, patients with stroke present an elevated fraction of activated positive cluster of differentiation 4 (CD4^+^) T cells in peripheral blood expressing interleukin-2 receptor alpha chain (CD25) or human leukocyte antigen-antigen D related (HLA-DR) when compared with controls [1,17,28]. Vogelgesang et al. [28] proposed the theory that HMGB1 acts as a link between brain tissue destruction by ischemic injury and the activation and type 1 T-helper (Th1) priming of T cells. They think it could be due to the similarity between the kinetics of serum HMGB1 and the kinetics observed for the absolute number of CD4^+^ T cells expressing HLA-DR [1,17,28]. Studies have further demonstrated that higher serum concentrations of HMGB1 are associated with the severity of neurologic impairment and with elevated serum levels of TNF-α and IL-1β [1,17,26,30].

## Cerebral HMGB1 receptors in IS

HMGB1 modulates numerous neuroinflammatory events via ligation to different cell surface receptors. The main receptors for HMGB1 investigated in brain injury are the receptor for advanced glycation end products (RAGE) and toll-like receptors (TLR-2 and TLR-4) as well as matrix metalloproteinases (MMPs). These receptors are ubiquitously expressed on central nervous system (CNS) resident microglia, astrocytes, and neurones [31,32].

### RAGE

There is a growing body of evidence that the RAGE and its ligands are involved in the pathogenesis of various disorders, including cardiovascular, neurodegenerative, inflammatory, and autoimmune disorders [33]. In the brain, RAGE is expressed on glial cells and neurones [34–36]. Biopsy samples of human patients with unilateral cerebral infarction have shown elevated levels of RAGE [37]. In murine experimental stroke, RAGE levels are elevated in the ischemic brain hemisphere a day after the stroke [37]. Remarkably, serum levels of sRAGE are elevated in stroke patients in the acute phase [38].

**Figure 1:**
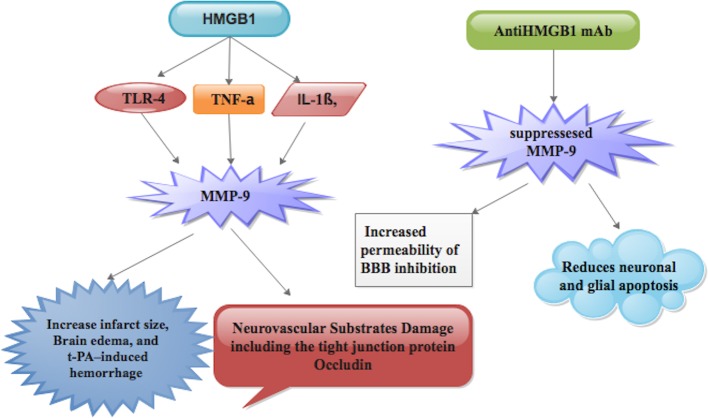
HMGB1 release during ischemic events interacts with TLR-2 and TLR-4 that is expression on monocytes via the adaptor protein MyD88 and elevates serum of TNF-α, IL-1β, and IL-6 levels, which leads to cerebral vessel occlusion Also, HMGB1 release and RAGE expression increase risk factors (HP, DM, HL), which contributes significantly to cerebral vessel occlusion during IS.

A hypertensive challenge has an early and sustained effect on RAGE up-regulation in brain vessels of the cortex and hippocampus in mice [2,39] and plasma sRAGE levels are decreased in patients with essential HT [2,40], which means that HMGB1 and RAGE contribute to the pathogenesis of HT, the key risk factor for stroke (Figure 1). Also, HMGB1 and RAGE contributed to the pathogenesis of HL (Figure 1). This was evidenced when cell culture experiments using monocyte cell culture (U937) showed the relocation of HMGB1 from the nucleus to the cytoplasm under HL stress [2,41]. Furthermore, HMGB1 and RAGE play a role in the progression of DM [2,42], which is one of the risk factors for stroke (Figure 1). A study has proven that hyperglycemia accelerates the release of HMGB1 and RAGE in human aortic endothelial cells [2,43]. Studies have further demonstrated that accumulation of advanced glycation end products (AGEs) in DM is associated with the pathogenesis of vascular and inflammatory cell complications that characterize DM [1,2,44]. Hu et al. [45] indicated that AGE-induced autophagy of vascular smooth muscle cells (VSMCs) contributes to the process of AGE-induced proliferation of rat VSMCs, which is related to atherosclerosis in DM [1,2]. Haraba et al. [41] observed an elevation in serum HMGB1 levels in golden Syrian hamsters with induced HL. They further observed that HMGB1 release and RAGE expression were elevated in cultures of U937 cells exposed to hyperlipidemic sera [1,41].

### TLRs in IS

TLRs are a category of receptors for a variety of molecular patterns originated from microorganisms but also identify tissue-released alarmins [1,46,47]. TLRs are a set of type 1 transmembrane proteins and have ten members in humans (TLR1–TLR10) and 11 members in mice (TLR1–TLR9 and TLR-11 and TLR-13) [1]. Architecturally, they consist of an extracellular domain, a transmembrane domain, and a cytoplasmic domain. The extracellular domain is involved in ligand recognition while the cytoplasmic domain is essential for signal transduction via association with the adaptor protein called myeloid differentiation primary response gene [88] (MyD88) (Figure 1) [1,48].

Brasier [49] has demonstrated that TLR-mediated signaling induces the secretion of proinflammatory cytokines and the proliferation of different cells. A study has shown that boosted expression of TLRs on CNS resident innate immune cells and subsequent release of toxic molecules are detrimental for neuronal survival and activation of TLRs has been linked to the progression of IS [1,50]. In stroke patients, increased TLR-2 and TLR-4 expression on monocytes is associated with higher serum levels of TNF-α, IL-1β, and IL-6 (Figure 1) [1,51]. TLR-2- and TLR-4-knockout mice showed less brain damage and neuronal deficits after 3 days of cerebral ischemia induced by MCAO [1,52,53]. Recently, Balosso et al. have shown that disulphide HMGB1 can increase *N*-Methyl-D-aspartic acid (NMDA)-induced neuronal cell death *in vitro* through its interaction with TLR-4 receptors [1,54]. The activation of microglia via TLR-4 induces amplification of neuronal cell loss and axonal damage after cerebral ischemia (Figure 1) [1,55].

## HMGB1 and MMPs in IS

**Figure 2:**
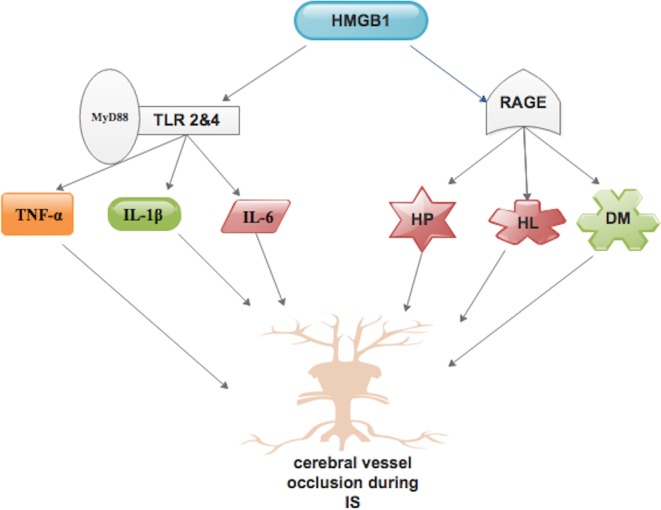
HMGB1 up-regulated MMP-9, which is mediated primarily through the TLR-4 HMGB1 induced cytokines such as TNF-α and IL-1β, which may promote MMP-9 up-regulation indirectly. Up-regulation of MMPs leads to increased infarct size, brain edema, and recombinant t-PA induced hemorrhage, which accelerates neurovascular substrate damage including the tight junction protein Occludin. Similarly, anti-HMGB1 mAb suppresses the activity of MMP-9, with an inhibition of the increased permeability of BBB, in ischemic brain area and reduces neuronal and glial apoptosis.

MMPs are proteases that can break down extracellular proteins, such as collagen, and are involved in extracellular matrix remodeling. These proteases are usually located in the cytosol in an inactivated state and are cleaved by plasmin or other MMPs to their active state [56,57]. Research has shown that MMP-9 has direct cytotoxic effects and can disrupt the BBB [58]. Studies have proven that the major source of MMP is microglia and they are released following ischemia, especially MMP-3 and MMP-9 [59,60]. Pfefferkorn and Rosenberg [61] demonstrated that acute MMP inhibition reduces infarct size, brain edema, and recombinant tissue plasminogen activator (t-PA) induced hemorrhage (Figure 2) in experimental stroke models. Further studies proved that mice deficient in MMP-9 or MMP-3 have reduced ischemic injury relative to wild-type controls [62,63]. However, persistent inhibition of MMPs after ischemia may have detrimental effects on functional recovery, because these proteases are important in neurovascular remodeling after stroke [64].

Sapojnikova et al. [65] indicated a strong correlation between MMP-9 secretion and HMGB1 levels, which was moreover linked with poor outcome in human IS. Qiu et al. demonstrated that HMGB1 up-regulated MMP-9 in cultured neurones and astrocytes, as well as in mouse brain (Figure 2) [66]. They stated that this phenomenon was mediated primarily through the TLR4, which was highly expressed in neuronal and astrocyte cultures as well as mouse brain (Figure 2). Furthermore, HMGB1-induced MMP-9 responses were mostly suppressed after blockade of TLR4 signaling. Therefore, HMGB1 constitutively expressed on TLR-4 receptors in adjacent brain, thus up-regulating MMP-9 and expanding neurovascular damage in ischemic brain injury. HMGB1 can also induce many other cytokines such as TNF-α and IL-1β, which may promote MMP-9 up-regulation indirectly (Figure 2) [66]. A potential source of elevated plasma MMP-9 and HMGB1 levels could be secretion from neutrophils, as MMP-9 is a neutrophil-derived blood MMP and a high quantity of HMGB1 characterizes these blood cells [65]. Activated MMP-9 could directly damage neurovascular substrates including the tight junction protein Occludin (Figure 2) [67]. Several studies have demonstrated that MMP-mediated degradation of Occludin is involved in the ischemia-induced BBB disruption [67–69]. Meanwhile therapeutically injected [70–72] and endogenous t-PA [73] have been implicated in the activation of MMP-9 during IS; HMGB1 may enhance the disruption of BBB structure through activation of MMPs via t-PA. Moreover, Liu et al. [7] indicated that anti-HMGB1 mAb suppresses the activity of MMP-9, with an inhibition of the increased permeability of BBB, in ischemic brain area (Figure 2). Inhibition of MMP-2 and MMP-9 reduces neuronal and glial apoptosis (Figure 2) [74].

## Atherosclerosis and HMGB1 in IS

Kalinina et al. [75] demonstrated that HMGB1 is essentially secreted by endothelial cells, smooth muscle cells and in CD68-positive macrophages situated close to the intima as well as in microvessels within the adventitia of human aorta. They further stated that active secretion of HMGB1 has been detected in areas adjacent to the necrotic core of atherosclerotic lesions [1,75]. Studies have shown that HMGB1 may be secreted from several cell types in the atherosclerotic plaque including smooth muscle cells, endothelial cells, foam cells, macrophages, and activated platelets [1,75–77]. HMGB1 when secreted, induces several inflammatory effects on endothelial cells, smooth muscle cells, and macrophages [17].

Studies have demonstrated that recombinant HMGB1 activates vascular endothelial cells initiating the release of intercellular adhesion molecule 1 (ICAM-1), vascular cell adhesion molecule 1 (VCAM-1), E-selectin, granulocyte colony stimulating factor (G-CSF), RAGE, TNF-α, monocyte chemotactic protein 1 (MCP-1), IL-8, plasminogen activator inhibitor 1, and t-PA [1,17,78–80]. Further studies have indicated that HMGB1 augments the formation of atherosclerotic plaques on smooth muscle cells as well as their proliferation, migration to the intimal layer, their release of more HMGB1 as well as C-reactive protein, and their secretion of MMP-2, MMP-3, and MMP-9 during IS [17,76,81]. The role of HMGB1 in the formation of atherosclerosis has been reported in apolipoprotein E deficient mice fed with a high-fat diet. Kanellakis et al. [82] reported that anti-HMGB1 neutralizing antibodies led to a dwindling of macrophage, dendritic cells (DC), and CD4+ T-cell accumulation in atherosclerotic lesions and decreased the secretion of VCAM-1 and MCP-1 [17,80]. They indicated that neutralization of monoclonal antibodies against HMGB1 lessens atherosclerosis by 55%. HMGB1 also plays a key role in the pathogenesis of thrombosis. Studies have shown that HMGB1 inhibits the anticoagulant protein C pathway via the thrombin–thrombomodulin complex, and stimulates release of tissue factor in monocytes *in vitro* [83,84]. Studies have further demonstrated that HMGB1 promotes development of microvascular thrombosis in rats [83,84].

## HMGB1 and angiogenesis in IS

The formation of new blood vessels in the late period of cerebral ischemia triggers a highly coupled neurorestorative process that mediates neurogenesis, synaptogenesis, and angiogenesis [1,85,86]. HMGB1 signaling can stimulate endothelial revival [79] and growth [87]. Researchers have shown that HMGB1 stimulates enlargement of endothelial progenitors, which boost angiogenesis; this outcome can be clogged by injection of HMGB1 siRNA 5 days post stroke [1,88,89]. HMGB1-mediated effect on angiogenesis may present a possible target for augmenting recovery several days after stroke onset. We therefore suggest that further studies are needed in this direction. Additionally, HMGB1 has been proven to accelerate neurite outgrowth and cell survival in neurones [1,90–92]. It has further been demonstrated that HMGB1 secreted by reactive astrocytes provides a signal for endothelial progenitor cell (EPC) mediated neurovascular remodeling, and this signaling between glial and vascular compartments may be critically significant for brain repair and stroke recovery [1,88]. Also, HMGB1 mediates cross-talk between reactive astrocytes and EPCs to promote functional recovery following focal cerebral ischemia [88].

## HMGB1 and edema formation in IS

Even though there are quite a lot of aquaporins (water channel proteins) expressed in the CNS, Aquaporin 4 (AQP4) is the major one in the brain, and is expressed in astroglial end feet around the surface of capillaries related to the BBB, glia limitans, and ependyma [93,94]. IS induces both immediate and delayed cell death, which is accompanied by a robust inflammatory response and cerebral edema that can exacerbate injury during reperfusion [94,95]. Brain edema is the main cause of death in patients with large infarction. AQP4, a water channel protein, is predominantly located in astroglial end feet [93,94] and has been shown to play an important role in water transport in the brain [94,96]. Also, AQP4 is considered to contribute to brain edema, particularly as AQP4-deficient mice have improved outcome following focal ischemic injury [94,97]. AQP4 may also contribute to cell adhesion [98] and migration [99], as well as neuroinflammation [94,100]. Interacting with other proinflammatory cytokines, extracellular HMGB1 aggravates inflammation and worsens injury in the ischemic brain, which further results in the up-regulation of aquaporins and exacerbation of brain edema. It is now clear that inhibition of HMGB1 is found to be neuroprotective for IS in rats [7,94]. We therefore propose that inhibition of HMGB1 could lead to reduction in AQP4 and hence reduction in brain edema.

## HMGB1 and neuroimmunological reactions in IS

Microglial cells are the resident macrophages of the brain and play an essential role as resident immunocomponent and phagocytic cells. It has been indicated that microglia activated under inflammation can transform into phacocytes and secrete a variety of mediators that are cytotoxic or cytoprotective [1,8]. Researchers have demonstrated that activated microglia after ischemia have the potential of secreting several proinflammatory cytokines such as TNF-α, IL-1, and IL-6, and nitric oxide ROS [1,101]. It has been proven that HMGB1 has essential roles for microglial activation minutes after cerebral ischemia [6] and may still be detected in microglia up to days after cerebral ischemia [24]. Although the infiltration of immune cells and the production of inflammatory mediators are conspicuous after IS, the direct activation of infiltrating immune cells is not achieved by HMGB1, which means that it may not be an ideal therapeutic target in the delayed phase of IS. Other researchers have proven that intravenous injection of neutralizing anti-HMGB1 mAb or intranasal delivery of HMGB1 siRNA provided a robust neuroprotection in the post-ischemic brain by antagonizing the proinflammatory function of HMGB1 [1,7,102].

## HMGB1 and hyperglycemia in IS

Studies have shown that glutamate, an excitatory amino acid, plays a central role in neuronal death [103,104]. Li et al. [105] demonstrated that hyperglycemia boosts extracellular glutamate accumulation in rats’ models of forebrain ischemia [104]. Qiu et al. [13] discovered that glutamate induces the secretion of HMGB1 from neurones *in vitro* [104]. Huang et al. therefore proposed that hyperglycemia could worsen brain ischemic damage by boosting the early extracellular secretion of HMGB1 [104]. They indicated that hyperglycemia augments the early secretion of HMGB1 from ischemic brain tissue, which led to increased infarct volume, neurological deficit, cerebral edema, and BBB disruption [104]. Initial research finding has shown that BBB was significantly disrupted 4 h after reperfusion in hyperglycemic rats [1,104,106,107]. Liu indicated that extracellular HMGB1 is involved in the disruption of BBB during the early phase of IS [7,104]. Disruption of BBB is a crucial event in the development of brain edema during the early stage of ischemic brain damage. Studies have shown that the development of brain edema is one of the most unfavorable ramification of stroke and is greatly boosted during hyperglycemic stroke [1,104,106,108].

## HMGB1 and post-ischemic brain recovery

Currently activated T lymphocytes are perceived as the key contributors to secondary tissue damage and facilitate the progression of stroke outcome. Shichita et al. [109] observed an important role of γ-δ T cells intervening in inflammatory brain damage after cerebral ischemia. Liesz et al. [110] demonstrated that inhibition of leukocyte-invasion to brain after cerebral ischemia decreases neuroinflammation and ischemic tissue injury. Studies have also shown that the regulatory T- and B-cell populations are neuroprotective after brain ischemia [1,111–113]. Further studies have indicated that IL-10 has been successfully used as a therapeutic mediator to reduce post-stroke secondary neuroinflammation [1,112,114–116]. Moreover, higher plasma levels of IL-6 are taken as a predictive marker of poor outcome in stroke patients [117].

**Figure 3:**
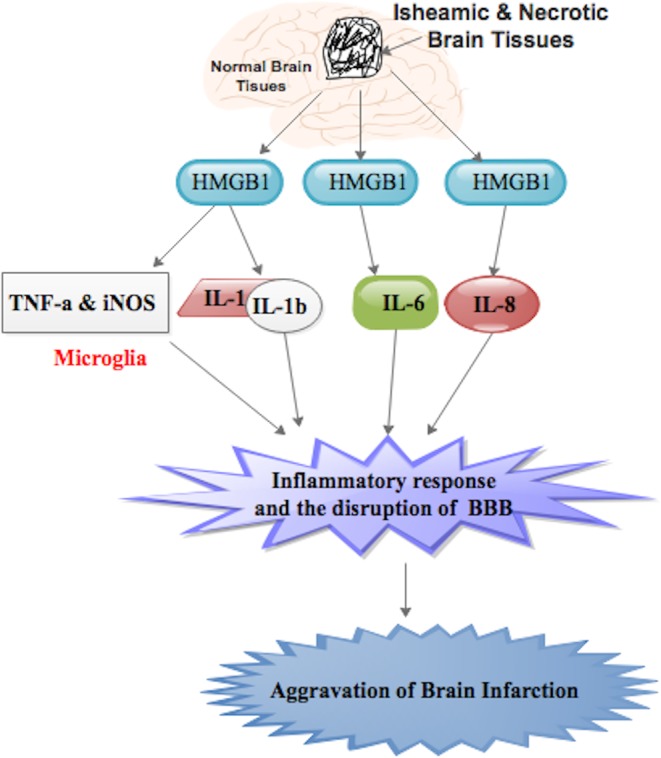
HMGB1 stimulates the production of IL-1, TNF-α, IL-6, and IL-8 and induces iNOS expression during ischemic brain damage Induction of iNOS and TNF-α occurs mainly in microglia. Induction of any of the factors above produces inflammatory response and the disruption of the BBB, leading to the aggravation of brain infarction.

HMGB1 has been reported to stimulate the production of IL-1, TNF-α, IL-6, and IL-8 and to induce iNOS expression during ischemic brain damage (Figure 3) [1,118,119]. Further studies have shown that the expression of TNF-α, iNOS, and IL-1β, which were all up-regulated in the post-ischemic brain, was reduced when HMGB1 was inhibited [1,6,18]. Also, neuroprotective effect was accompanied by a reduction in the release of HMGB1 into the extracellular space and in the related inflammatory factors TNF-α, iNOS, IL-1β, and IL-6, both in the brain and serum of rats. The induction of iNOS and TNF-α following an ischemic insult was reported to occur mainly in microglia (Figure 3). Thus, it is likely that HMGB1 activates microglia in the brain, leading to the up-regulation of iNOS and TNF-α expression (Figure 3). The induction of iNOS and TNF-α has been reported to be involved in the inflammatory response and the disruption of the BBB, leading to the aggravation of brain infarction (Figure 3) [1,120,121]. The regulation of any one of these factors has been postulated to reduce ischemic injury.

Apoptosis is tightly regulated by the mitogen-activated protein kinase (MAPK) family, and the c-Jun N-terminal kinases (JNKs), extracellular signal-regulated kinase 1/2 (ERK1/2), and p38 members of this family have been demonstrated to be activated in ischemic reperfusion injury (I/R) [1,122]. MAPKs play important roles in transducing signals by phosphorylating intracellular enzymes, transcription factors, and cytosolic proteins involved in apoptosis and inflammatory cytokine production. Sustained MAPK activation has been shown to be associated with neuronal cell death/apoptosis following IS, and the inhibition of this pathway is neuroprotective [1,122].

## HMGB1 and therapeutic potentials of stroke medications

Therapeutic choices for acute stroke are still limited notwithstanding significant advances in the understanding of the pathophysiology of cerebral ischemia [123]. Statins have proven to attenuate the effects of HMGB1 on endothelial cells in two experimental studies. Yang et al. demonstrated that atorvastatin is able to inhibit endothelial activation *in vitro* upon HMGB1 stimulation in a dose-dependent manner [17,124]. They incubated endothelial cells with 10 μM of atorvastatin and noticed that expression of ICAM-1 and E-selectin are reduced and inhibited HMGB1-stimulated leukocyte adhesion to endothelial cells. Furthermore, atorvastatin similarly suppressed HMGB1-induced TLR-4 expression and NF-κB nuclear translocation in endothelial cells [17,124]. Wang et al. further observed that atorvastatin had neuroprotective effect, and this effect is related to the decreased expression of HMGB1, its receptors (TLR-4 and RAGE), NF-κB in the acute phase of cerebral ischemia [17,125]. They therefore proposed that down-regulation of HMGB1-induced NF-κB activation pathway might be a potential mechanism of atorvastatin’s neuroprotection in cerebral ischemia [17,125]. Haraba et al. [41] observed that fluvastatin reduced serum HMGB1 levels by 38.2% and led to a 1.46-fold reduction in HMGB1 mRNA expression in lung tissue [41]. The blockade of HMGB1 signaling with shRNA in the post-ischemic brain suppressed infarct size, microglial activation, and induction of proinflammatory mediators [6]. Parkkinen et al. [126] have demonstrated that HMGB1 has affinity for t-PA and accelerates its proteolytic activity.

Xiong et al. [94] have also demonstrated that administration of probenecid reduced infarct size, inhibited HMGB1 release from neurones, and down-regulated AQP4 expression in the cortical penumbra 48 h post stroke. They also observed that probenecid inhibits the activation of astrocytes and up-regulates AQP4 protein expression 48 h post stroke, which results in the attenuation of cerebral edema following focal ischemia. Because of its critical role in brain edema formation, AQP4 is regarded as an attractive therapeutic target for various brain disorders, including brain trauma, tumor, hydrocephalus, neuroinflammation, and focal cerebral ischemic injury [94,127]. Probenecid, as an inhibitor of Panx1 channels, inhibits ATP release induced by Panx1 activation [128,129], blocks inflammasome formation, and attenuates caspase 1 cleavage [130], thereby alleviating neuronal death and reducing brain inflammation and cerebral edema [94].

Administration of anti-HMGB1 neutralizing antibodies in experimental models of MCAO/reperfusion showed a significant decrease in infarct size and an improvement in neurologic deficits in treated rats. Anti-HMGB1 antibodies also prevented the increase in permeability of the BBB protecting the recipient from brain edema, inhibited activation of microglia and release of TNF-α and iNOS, while suppressing the activity of MMP-9 [1,7,12]. Additionally, the administration of numerous agents such as atorvastatin, minocycline, edaravone, cannabinol, niaspan, and Tricin 7-glucoside was also shown to inhibit HMGB1 expression in brain ischemic tissue during the acute phase after stroke in experimental models of MCAO, so alleviating cerebral injury [1,125,131–135].

Tanshinone II A (Tan II A) effectively decreased neurologic impairment and tissue injury under cerebral ischemic conditions. This effect may be through down-regulation of HMGB1/HMGB1 receptors (TLR4 and RAGE)/NF-κB activation pathway, up-regulation of claudin-5 expression, and reduction in extravascular IgG. HMGB1 relation with NF-κB activation pathway may be one of Tan II A’s effective therapeutic targets for cerebral ischemia. Tan II A inhibits HMGB1 translocation from nucleus to cytoplasm and/or extracellular space in the cortical peri-infarct regions [136].

Glycyrrhizin (GL) could be a new therapeutic possibility for the treatment of IS. Gong et al. [122] demonstrated that pretreatment with GL blocked and inhibited the extracellular cytokine activity of HMGB1 and explored the protective effect on I/R-induced apoptosis through the blockage of the JNK and p38-mediated pathways in rats *in vivo*. They stated that pretreatment with GL alleviated apoptosis injury resulting from cerebral I/R, and this was at least partly due to the inhibition of cytochrome *c* release and caspase 3 activity. Gwak et al. [137] further demonstrated that GL exhibited an anti-apoptotic effect by preventing HMGB1-induced cytochrome *c* release and caspase 3 activation *in vitro* in Huh-BAT cells (Korean Cell Line Bank, Seoul, Korea). Although the anti-apoptotic effect of GL has been linked with HMGB1 inhibition and caspase-dependent cytochrome *c* release, it is still not very clear how GL regulates caspase-dependent cytochrome *c* release through the inhibition of HMGB1. Some authors are of the view that GL may modulate the activity of a particular kinase that contributes to cytochrome *c* translocation and is involved in I/R-induced HMGB1-dependent apoptosis [122].

## Conclusion

IS leads to the disruption of cerebral supply with nutrients and oxygen leading to permanent necrotic brain tissue destruction in the affected area of the respective terminal artery. HMGB1 presents dual effects after ischemic injury in the brain. HMGB1 acts as a proinflammatory mediator in the acute phase that amplifies damage in ischemic tissue through the activation of microglia, enhancement of inflammation, and increase in permeability of the BBB. In contrast, in the late phase after ischemic injury HMGB1 contributes to the recovery and remodeling process stimulating neurovascular repair mainly by astrocytes in the affected brain. We therefore suggest that more research with both animal and human models is needed to further confirm that HMGB1 has therapeutic potentials since initial studies proved that it promotes neurovascular repair and remodeling after stroke.

## Abbreviations

AGE: advanced glycation end product
AQP4: aquaporin 4
BBB: blood–brain barrier
CNS: central nervous system
DAMP: danger-associated molecular pattern
DM: diabetes mellitus
EPC: endothelial progenitor cell
GL: glycyrrhizin
HL: hyperlipidemia
HLA-DR: human leukocyte antigen-antigen D related
HMGB1: high mobility group box 1
HT: hypertension
ICAM-1: intercellular adhesion molecule 1
IL-1β: interleukin-1β
iNOS: inducible nitric oxide synthase
IS: ischemic stroke
JNK: c-Jun N-terminal kinase
MAPK: mitogen-activated protein kinase
MCAO: middle cerebral artery occlusion
MCP-1: monocyte chemotactic protein 1
MMP: matrix metalloproteinase
NF-κB: nuclear factor κ-light-chain-enhancer of activated B cell
RAGE: receptor for advanced glycation end product
RNS: reactive nitrogen species
ROS: reactive oxygen species
sRAGE: soluble RAGE
t-PA: tissue plasminogen activator
Tan II A: tanshinone II A
TLR: toll-like receptor
TNF-α: tumor necrosis factor α
VCAM-1: vascular cell adhesion molecule 1
VSMC: vascular smooth muscle cell
